# Supplementary material for: Autoantibodies neutralizing type I IFNs in 40% of patients with WNV encephalitis in seven new cohorts
Source: J Hum Immun. 2026 Mar 13;2(3):e20250189. doi: 10.70962/jhi.20250189 (PMC12984024; doi:10.70962/jhi.20250189)
Supplement: Table S1 — shows prevalence of WNVIC, WNVF, WNVD, or WNND of subjects carrying auto-Abs neutralizing at least 1 IFN-I by WNV lineage (WNV-1 or WNV-2). [file jhi_20250189_tables1.docx]

**Supplemental table 1 (table S1). Prevalence of WNVIC, WNVF, WNVD, or WNND of subjects carrying auto-Abs neutralizing at least 1 IFN-I by WNV lineage (WNV-1 or WNV-2).**

| **WNV group** | **WNV-1** | | **WNV-2** | | |
| --- | --- | --- | --- | --- | --- |
|  | **Detected Cases** | **Anti-IFN-α2 (100 pg/ml) and/or anti-IFN-ω (100 pg/ml) and/or anti-IFN-β (1 ng/ml)** | **Detected Cases** | **Anti-IFN-α2 (100 pg/ml) and/or anti-IFN-ω (100 pg/ml) and/or anti-IFN-β (1 ng/ml)** | **P value** |
| WNVIC | 16/66 (24%) | 0/16 (0%) | 50/66 (76%) | 1/50 (2%) |  |
| WNVF | 21/84 (25%) | 1/21 (5%) | 63/84 (75%) | 6/63 (9%) |  |
| WNVD | 69/230 (30%) | 23/69 (33%) | 161/230 (70%) | 58/161 (36%) |  |
| WNND | 56/160 (35%) | 19/56 (34%) | 104/160 (65%) | 48/104 (46%) | 0.81 |
| WNE | 35/56 (63%) | 4/19 (21%) | 53/104 (51%) | 29/53 (55%) | 0.02 |
| WNM | 19/56 (34%) | 14/35 (40%) | 31/104 (30%) | 13/31 (42%) |  |
| AFP | 2/56 (4%) | 1/2 (50%) | 1/104 (1%) | 0/1 (0%) |  |
| UNS | 0/56 (0%) | — | 19/104 (18%) | 6/19 (32%) |  |

WNVIC: West Nile virus infected controls; WNVF: West Nile virus fever; WNVD: West Nile virus disease; WNND: West Nile virus neurological disease; WNE: WNV encephalitis; WNM: WNV meningitis; AFP: acute flaccid paralysis; UNS: unspecified neurological syndrome

Counts or frequency (%)
