# Supplementary material for: Autoantibodies neutralizing type I IFNs in 40% of patients with WNV encephalitis in seven new cohorts
Source: J Hum Immun. 2026 Mar 13;2(3):e20250189. doi: 10.70962/jhi.20250189 (PMC12984024; doi:10.70962/jhi.20250189)
Supplement: Table S2 — shows risk of WNVIC, WNVF, WNVD, or WNND of subjects carrying auto-Abs neutralizing at least one IFN-I by sex. [file jhi_20250189_tables2.docx]

**Supplemental table 2 (table S2). Risk of WNVIC, WNVF, WNVD, or WNND of subjects carrying auto-Abs neutralizing at least 1 IFN-I by sex.**

| **WNV group** | | **New Cohort** | | | | **New + Old Cohort** | | | |
| --- | --- | --- | --- | --- | --- | --- | --- | --- | --- |
|  |  | **Males** | **Females** | **OR [95%CI]** | **P value** | **Males** | **Females** | **OR [95%CI]** | **P value** |
| WNVIC |  | 1/27 [4%] | 0/5 [0%] | — | 1.0 | 3/114 [3%] | 0/32 [0%] | — | 1.0 |
| WNVF |  | 1/33 [3%] | 0/27 [0%] | — | 1.0 | 11/88 [13%] | 5/80 [6%] | — | 0.17 |
| WNVD |  | 60/147 [41%] | 19/76 [25%] | 2.07 [1.13– 3.89] | 0.02 | 172/433 [40%] | 63/231 [27%] | 1.75 [1.24– 2.48] | 1.7×10⁻³ |
| WNND | | 53/130 [41%] | 17/68 [25%] | 2.06 [1.09– 4.04] | 0.03 | 149/362 [41%] | 56/184 [30%] | 1.59 [1.09– 2.33] | 0.02 |

WNVIC: West Nile virus infected controls; WNVF: West Nile virus fever; WNVD: West Nile virus disease; WNND: West Nile virus neurological disease; WNE: WNV encephalitis; WNM: WNV meningitis; AFP: acute flaccid paralysis; UNS: unspecified neurological syndrome

Counts or frequency (%)
