# Supplementary material for: Autoantibodies neutralizing type I IFNs in 40% of patients with WNV encephalitis in seven new cohorts
Source: J Hum Immun. 2026 Mar 13;2(3):e20250189. doi: 10.70962/jhi.20250189 (PMC12984024; doi:10.70962/jhi.20250189)
Supplement: Table S3 — shows risk of neuroinvasive disease and encephalitis for subjects carrying auto-Abs neutralizing specific sets of type I IFNs, relative to the general population, with adjustment for age and sex and risk by age group. [file jhi_20250189_tables3.docx]

**Supplemental table 3 (table S3). Risk of neuroinvasive disease a and encephalitis for subjects carrying auto-Abs neutralizing specific sets of type I IFNs, relative to the general population, with adjustment for age and sex and risk by age group.**

| **Anti-type I IFN auto-Ab (amount of type I IFN neutralized, in plasma diluted 1:10)** |  | **New cohort** | | **Overall cohort** | |
| --- | --- | --- | --- | --- | --- |
|  | **WNV group** | **OR [95%CI]** | **P value** | **OR [95%CI]** | **P value** |
| Anti-IFN-ω (100 pg/ml) regardless of other type I IFNs neutralization | WNND (All) | 21.7 [15.4-30.6] | < 10^-16^ | 25 [19.8-31.7] | < 10^-16^ |
|  | WNND ≤ 65 | 24.3 [12-49] | < 10^-16^ | 27.4 [17.1-43.7] | < 10^-16^ |
|  | WNND > 65 | 29 [18.9-44.6] | < 10^-16^ | 28.8 [21.6-38.5] | < 10^-16^ |
| Anti-IFN-α2 (100 pg/ml) regardless of other type I IFNs neutralization | WNND (All) | 25 [17.4-35.8] | < 10^-16^ | 29.4 [23-37.6] | < 10^-16^ |
|  | WNND ≤ 65 | 86.6 [41.4-181.2] | < 10^-16^ | 80.1 [44.7-143.6] | < 10^-16^ |
|  | WNND > 65 | 22.5 [14.7-34.5] | < 10^-16^ | 26.2 [19.8-34.7] | < 10^-16^ |
| Anti-IFN-α2 (100 pg/ml) and/or anti-IFN-ω (100 pg/ml) and/or anti-IFN-β (10 ng/ml) | WNND (All) | 16.8 [12.1-23.4] | < 10^-16^ | 18.6 [14.9-23.2] | < 10^-16^ |
|  | WNND ≤ 65 | 24.4 [13.7-43.7] | < 10^-16^ | 20.7 [13.7-31.3] | < 10^-16^ |
|  | WNND > 65 | 18.1 [12-27.3] | < 10^-16^ | 20.2 [15.4-26.6] | < 10^-16^ |
| Anti-IFN-α2 (100 pg/ml) and/or anti-IFN-ω (100 pg/ml) regardless of anti-IFN-β | WNND (All) | 18.1 [13.1-25.1] | < 10^-16^ | 20.2 [16.2-25.0] | < 10^-16^ |
|  | WNND ≤ 65 | 31.9 [17.6-57.8] | < 10^-16^ | 26.7 [17.4-40.8] | < 10^-16^ |
|  | WNND > 65 | 20.2 [13.4-30.4] | < 10^-16^ | 22.3 [17.1-29.1] | < 10^-16^ |
| Anti-IFN-α2 (100 pg/ml) and anti-IFN-ω (100 pg/ml) regardless of anti-IFN-β | WNND (All) | 42.2 [27.8-63.9] | < 10^-16^ | 53.6 [39.4-72.9] | < 10^-16^ |
|  | WNND ≤ 65 | 378.8 [96.3-1490] | 8.9 x 10^-16^ | 383.1 [131-1120.4] | < 10^-16^ |
|  | WNND > 65 | 40.6 [25.4-64.7] | < 10^-16^ | 42.9 [30.8-59.7] | < 10^-16^ |
| Anti-IFN-ω (10 ng/ml) regardless of other type I IFNs neutralization | WNND (All) | 48.7 [32.5-73.1] | < 10^-16^ | 56.8 [42.7-75.6] | < 10^-16^ |
|  | WNND ≤ 65 | 100.6 [40.2-251.6] | 2.6 x 10^-11^ | 134.7 [74.5-243.6] | < 10^-16^ |
|  | WNND > 65 | 45.8 [29.3-71.7] | 9.2 x 10^-12^ | 44.5 [32.4-60.9] | < 10^-16^ |
| Anti-IFN-α2 (10 ng/ml) regardless of other type I IFNs neutralization | WNND (All) | 51.6 [34.8-76.4] | < 10^-16^ | 65.9 [50.2-86.5] | < 10^-16^ |
|  | WNND ≤ 65 | 273.4 [119.9-623.0] | < 10^-16^ | 245.3 [129.9-463.4] | < 10^-16^ |
|  | WNND > 65 | 39.5 [25.4-61.4] | < 10^-16^ | 49.4 [36.8-66.4] | < 10^-16^ |
| Anti-IFN-α2 (10 ng/ml) and/or anti-IFN-ω (10 ng/ml) and/or anti-IFN-β (10 ng/ml) | WNND (All) | 28 [19.1-41] | < 10^-16^ | 32.8 [24.9-43.3] | < 10^-16^ |
|  | WNND ≤ 65 | 46.4 [21.6-99.6] | 2.2 x 10^-14^ | 45.7 [26.1-79.9] | < 10^-16^ |
|  | WNND > 65 | 27.7 [17.7-43.4] | < 10^-16^ | 31.1 [22.5-42.9] | < 10^-16^ |
| Anti-IFN-α2 (10 ng/ml) and/or anti-IFN-ω (10 ng/ml) regardless of anti-IFN-β | WNND (All) | 36.9 [25.4-53.5] | < 10^-16^ | 46.2 [36.1-59.2] | < 10^-16^ |
|  | WNND ≤ 65 | 131.2 [63.2-272.4] | < 10^-16^ | 122.3 [72.6-206.1] | < 10^-16^ |
|  | WNND > 65 | 30.6 [20.0-46.7] | < 10^-16^ | 37.0 [28.1-48.7] | < 10^-16^ |
| Anti-IFN-α2 (10 ng/ml) and anti-IFN-ω (10 ng/ml) regardless of anti-IFN-β | WNND (All) | 101.3 [63.3-162.1] | < 10^-16^ | 123.5 [85.6-178.3] | < 10^-16^ |
|  | WNND ≤ 65 | 442 [128.6-1518.6] | 2.5 x 10^-14^ | 602 [224.2-1616.2] | < 10^-16^ |
|  | WNND > 65 | 82.9 [50.2-137] | < 10^-16^ | 84.6 [57.6-124.3] | < 10^-16^ |
| Anti-IFN-α2 (10 ng/ml) and anti-IFN-ω (100 pg/ml) regardless of anti-IFN-β | WNND (All) | 59.0 [34.8-100.2] | < 10^-16^ | 83.1 [53.8-128.5] | < 10^-16^ |
|  | WNND ≤ 65 | 1616.4 [84.1-31074.1] | 2.3 x 10^-13^ | 1841.1 [109.5-30940.6] | < 10^-16^ |
|  | WNND > 65 | 48.6 [27.9-84.8] | < 10^-16^ | 58.4 [37.5-90.9] | < 10^-16^ |
| Anti-IFN-α2 (10 ng/ml) and anti-IFN-ω (10 ng/ml) and anti-IFN-β (10 ng/ml) | WNND (All) | 243.9 [12.5-4744.1] | 2.9 x 10^-6^ | 142.0 [7.9-2535.1] | 1.1 x 10^-7^ |
|  | WNND ≤ 65 | 113.1 [4.5-2826.3] | 3 x 10^-3^ | 46.3 [1.9-1146.1] | 1.0 x 10^-2^ |
|  | WNND > 65 | 159.5 [7.5-3412.3] | 2 x 10^-4^ | 110.4 [6-2020.6] | 2.2 x 10^-6^ |
| Anti-IFN-ω (100 pg/ml) regardless of other type I IFNs neutralization | WNE (All) | 23.7 [16.3-34.6] | < 10^-16^ | 22.8 [17.4-29.8] | < 10^-16^ |
|  | WNE ≤ 65 | 29.7 [13.1-67.3] | 9.2 x 10^-10^ | 27.9 [15.8-49.1] | < 10^-16^ |
|  | WNE > 65 | 29.5 [18.8-46.4] | < 10^-16^ | 25.6 [18.6-35.4] | < 10^-16^ |
| Anti-IFN-α2 (100 pg/ml) regardless of other type I IFNs neutralization | WNE (All) | 26.1 [17.6-38.7] | < 10^-16^ | 25.1 [19.0-33.2] | < 10^-16^ |
|  | WNE ≤ 65 | 93.5 [40.5-215.9] | < 10^-16^ | 73.2 [37.5-143.1] | < 10^-16^ |
|  | WNE > 65 | 23.3 [14.9-36.5] | < 10^-16^ | 22.5 [16.4-30.8] | < 10^-16^ |
| Anti-IFN-α2 (100 pg/ml) and/or anti-IFN-ω (100 pg/ml) and/or anti-IFN-β (10 ng/ml) | WNE (All) | 17.4 [12.1-25.1] | < 10^-16^ | 16.4 [12.7-21.2] | < 10^-16^ |
|  | WNE ≤ 65 | 25.6 [12.7-51.3] | 1.1 x 10^-12^ | 19.5 [11.8-32.4] | < 10^-16^ |
|  | WNE > 65 | 18.5 [11.9-28.7] | < 10^-16^ | 17.3 [12.7-23.5] | < 10^-16^ |
| Anti-IFN-α2 (100 pg/ml) and/or anti-IFN-ω (100 pg/ml) regardless of anti-IFN-β | WNE (All) | 18.8 [13.1-27.0] | < 10^-16^ | 17.7 [13.8-22.8] | < 10^-16^ |
|  | WNE ≤ 65 | 33.5 [16.5-67.9] | 5.5 x 10^-14^ | 25.1 [14.9-42.0] | < 10^-16^ |
|  | WNE > 65 | 20.6 [13.3-31.9] | < 10^-16^ | 19.1 [14.2-25.8] | < 10^-16^ |
| Anti-IFN-α2 (100 pg/ml) and anti-IFN-ω (100 pg/ml) regardless of anti-IFN-β | WNE (All) | 46.4 [29.8-72.2] | < 10^-16^ | 46.4 [33.1-65.0] | < 10^-16^ |
|  | WNE ≤ 65 | 705.6 [138.6-3592.6] | 3.3 x 10^-15^ | 420.7 [128.5-1377.3] | < 10^-16^ |
|  | WNE > 65 | 42.1 [25.8-68.6] | < 10^-16^ | 38.3 [26.6-55] | < 10^-16^ |
| Anti-IFN-ω (10 ng/ml) regardless of other type I IFNs neutralization | WNE (All) | 50.4 [32.6-78.1] | < 10^-16^ | 48.6 [35.2-67.2] | < 10^-16^ |
|  | WNE ≤ 65 | 142.9 [51.7-394.8] | 1.5 x 10^-10^ | 142.8 [70.1-291] | < 10^-16^ |
|  | WNE > 65 | 45.8 [28.5-73.5] | < 10^-16^ | 39.3 [27.7-55.8] | < 10^-16^ |
| Anti-IFN-α2 (10 ng/ml) regardless of other type I IFNs neutralization | WNE (All) | 52.0 [34.1-79.5] | < 10^-16^ | 55.6 [40.9-75.5] | < 10^-16^ |
|  | WNE ≤ 65 | 304.8 [118.7-782.8] | < 10^-16^ | 268.9 [128.8-561.5] | < 10^-16^ |
|  | WNE > 65 | 41.3 [26-65.6] | < 10^-16^ | 42.5 [30.6-59] | < 10^-16^ |
| Anti-IFN-α2 (10 ng/ml) and/or anti-IFN-ω (10 ng/ml) and/or anti-IFN-β (10 ng/ml) | WNE (All) | 29.1 [19.2-44.0] | < 10^-16^ | 28.2 [20.6-38.4] | < 10^-16^ |
|  | WNE ≤ 65 | 54.5 [22.6-131.6] | 1.3 x 10^-11^ | 44.3 [22.8-86.2] | < 10^-16^ |
|  | WNE > 65 | 27.8 [17.4-44.6] | < 10^-16^ | 26.4 [18.6-37.6] | < 10^-16^ |
| Anti-IFN-α2 (10 ng/ml) and/or anti-IFN-ω (10 ng/ml) regardless of anti-IFN-β | WNE (All) | 36.7 [24.4-55.1] | < 10^-16^ | 38.7 [29.1-51.4] | < 10^-16^ |
|  | WNE ≤ 65 | 155.9 [66.7-364.5] | 1.1 x 10^-15^ | 129.9 [69.5-243.1] | < 10^-16^ |
|  | WNE > 65 | 30.9 [19.7-48.3] | < 10^-16^ | 31.3 [22.9-42.7] | < 10^-16^ |
| Anti-IFN-α2 (10 ng/ml) and anti-IFN-ω (10 ng/ml) regardless of anti-IFN-β | WNE (All) | 106.7 [65.0-175.0] | < 10^-16^ | 105.8 [71.2-157.3] | < 10^-16^ |
|  | WNE ≤ 65 | 554.5 [145.9-2107.5] | 6.8 x 10^-13^ | 649.6 [217.9-1937] | < 10^-16^ |
|  | WNE > 65 | 87 [51.6-146.8] | < 10^-16^ | 76.1 [50.3-115.1] | < 10^-16^ |
| Anti-IFN-α2 (10 ng/ml) and anti-IFN-ω (100 pg/ml) regardless of anti-IFN-β | WNE (All) | 65.9 [38.1-114.2] | < 10^-16^ | 71.8 [45.3-113.7] | < 10^-16^ |
|  | WNE ≤ 65 | 2597.5 [117.1-57597.3] | 3.9 x 10^-12^ | 2218.4 [125.1-39337.7] | < 10^-16^ |
|  | WNE > 65 | 54 [30.4-95.8] | < 10^-16^ | 52.1 [32.6-83.3] | < 10^-16^ |
| Anti-IFN-α2 (10 ng/ml) and anti-IFN-ω (10 ng/ml) and anti-IFN-β (10 ng/ml) | WNE (All) | 218.5 [10.3-4629.5] | 6.0 x 10^-5^ | 128.0 [6.8-2414.0] | 5.1 x 10^-6^ |
|  | WNE ≤ 65 | 147.6 [5.9-3717.3] | 2 x 10^-3^ | 63.2 [2.5-1569] | 8 x 10^-3^ |
|  | WNE > 65 | 80.7 [3.3-2004.6] | 5 x 10^-3^ | 74.8 [3.8-1455.8] | 1.6 x 10^-4^ |

WNND: West Nile neuroinvasive disease, a subgroup of WNVD; WNE: West Nile Encephalitis, a subgroup of WNND. ≤65 and >65 indicate age cut-offs; anti-IFN-ω and anti-IFN-α2 indicate auto-Abs neutralizing IFN-ω or IFN-α2, respectively, regardless of their effects on other IFNs
